# A Millifluidic Study of Cell-to-Cell Heterogeneity in Growth-Rate and Cell-Division Capability in Populations of Isogenic Cells of *Chlamydomonas reinhardtii*

**DOI:** 10.1371/journal.pone.0118987

**Published:** 2015-03-11

**Authors:** Shima P. Damodaran, Stephan Eberhard, Laurent Boitard, Jairo Garnica Rodriguez, Yuxing Wang, Nicolas Bremond, Jean Baudry, Jérôme Bibette, Francis-André Wollman

**Affiliations:** 1 Laboratoire de Colloïdes et Matériaux Divisés, Institute of Chemistry, Biology and Innovation ESPCI ParisTech/CNRS UMR 8231/PSL* Research University, Paris, France; 2 Laboratoire de Physiologie Membranaire et Moléculaire du Chloroplaste, Institut de Biologie Physico-Chimique, UMR CNRS/UPMC 7141, Paris, France; 3 Optical Science & Engineering Research Center, Department of Physics and Astronomy, Shanghai Jiao Tong University, Shanghai, China; University of Hyderabad, INDIA

## Abstract

To address possible cell-to-cell heterogeneity in growth dynamics of isogenic cell populations of *Chlamydomonas reinhardtii*, we developed a millifluidic drop-based device that not only allows the analysis of populations grown from single cells over periods of a week, but is also able to sort and collect drops of interest, containing viable and healthy cells, which can be used for further experimentation. In this study, we used isogenic algal cells that were first synchronized in mixotrophic growth conditions. We show that these synchronized cells, when placed in droplets and kept in mixotrophic growth conditions, exhibit mostly homogeneous growth statistics, but with two distinct subpopulations: a major population with a short doubling-time (fast-growers) and a significant subpopulation of slowly dividing cells (slow-growers). These observations suggest that algal cells from an isogenic population may be present in either of two states, a state of restricted division and a state of active division. When isogenic cells were allowed to propagate for about 1000 generations on solid agar plates, they displayed an increased heterogeneity in their growth dynamics. Although we could still identify the original populations of slow- and fast-growers, drops inoculated with a single progenitor cell now displayed a wider diversity of doubling-times. Moreover, populations dividing with the same growth-rate often reached different cell numbers in stationary phase, suggesting that the progenitor cells differed in the number of cell divisions they could undertake. We discuss possible explanations for these cell-to-cell heterogeneities in growth dynamics, such as mutations, differential aging or stochastic variations in metabolites and macromolecules yielding molecular switches, in the light of single-cell heterogeneities that have been reported among isogenic populations of other eu- and prokaryotes.

## Introduction

Most of the experiments usually performed with unicellular organisms use liquid bulk cultures, streaks or colonies, which reflect the summed and averaged behavior of about 10^6^–10^9^ individual cells from a same batch. The cells constituting a given batch are assumed to have close enough behaviors to be well represented by the overall behavior of the bulk culture itself. The underlying rationale is that a phenotype results from a genotype expressed in a given environment. In most cases, care is taken to inoculate a bulk culture with isogenic cells (same genotype), which, being placed in identical growth conditions (same environment), should display identical phenotypes. When limited genetic heterogeneity may exist at the start of a culture, it is commonly assumed that during growth in bulk conditions this heterogeneity becomes lower and disappears over time, owing to the prevalence of the genotype the most appropriate to the growth conditions used in that particular experiment, which would outcompete other genetic variants.

However, as early as 1932, it was shown that individual bacteria originating from young liquid cultures, *i*.*e*. presumably representing an isogenic population in current terms, display considerable diversity in their growth-rate, with slow- and fast-growing bacteria, as well as many intermediate growth phenotypes [1]. Later on, stochastic variations in phenotypic traits in populations of isogenic cells of *E*. *coli* have been observed, notably for expression of the lactose operon [2], or chemotaxis and swimming behavior [3]. Other well-known examples of bacterial cell-to-cell heterogeneity include the triggering of sporulation [4] and the establishment of genetic competence during the transition to stationary phase, which develops only within a minor subpopulation of bacteria that stop growing and become capable of transformation, because of the stochastic activation of a master regulator [5]. Cell-to-cell variability in microbial populations has since been actively studied (reviewed in [6,7]). Stochastic gene expression in clonal populations of both pro- and eukaryotic cells has been shown to result from “intrinsic noise”, which arises from inherent variabilities in biochemical processes of gene expression and in metabolic or signaling pathways, and from “extrinsic noise”, due to environmental changes, as well as to fluctuations in the concentration of other cellular components, such as regulatory proteins and polymerases for example [8–10]. Small changes in the concentration of these molecules can lead to significant cell-to-cell heterogeneity (reviewed in [11]), as a result of molecular switches, related to the activation/repression status of regulatory pathways, ultimately driving them to different phenotypes and hence contributing to the generation of distinct subpopulations [12]. In isogenic clonal mammalian cell populations, dramatic phenotypical cell-to-cell heterogeneities have been shown to be ubiquitous, and play important biological roles in cell structure, morphology, cell-fate decision, cell division, cell death and many other important cellular processes (reviewed in [8,13,14]), leading authors to stress that beyond just being “noise”, these phenomena play pivotal biological roles in many organisms (reviewed in [11,15]). The most studied unicellular eukaryotic model for cell-to-cell heterogeneity is the yeast *Saccharomyces cerevisiae* in which cell-fate decisions relating to growth dynamics (divide, not divide, grow, stop to grow) can be stochastically different between isogenic cells. These stochastic differences have been correlated to fluctuations in metabolites and in differing capacities of individual cells to transmit signals through signaling pathways [16]. Another major source of cell-to-cell heterogeneity in *S*. *cerevisiae* stems from its asymmetric cell division, that is associated with differential aging of cells among isogenic populations [17]. Replicative aging (replicative life-span, marked by a decrease in cell-division capacity, due, among others, to telomere shortening [18,19]), chronological aging (survival time of non-dividing cells, due, among others, to cellular damage [20–22]) as well as to uneven distribution of cellular components between the two daughter cells, all contribute to the generation of an “older” and a “younger” daughter cell, the older one ultimately stopping to divide [23–25]. It is of note that, with the advent of multiple methods enabling the study of phenotypic traits at the single-cell level, including milli- and micro-fluidic methods, alginate hydrogel beads and flow cytometry, the existence of common and widespread cell-to-cell heterogeneity has largely been verified, with numerous reports highlighting cell-to-cell phenotypical variations in isogenic populations of both pro- and eukaryotic cells [6,26–39].

Studies of cell-to-cell heterogeneity in eukaryotic microalgae are of growing interest, because these organisms are being used as model systems for the studies of many fundamental biological processes [40], as well as in many commercial, industrial and biological applications (reviewed in [41,42]). For example, as the depletion of fossil fuels demands alternative renewable energy sources, there has been a recent and increased interest to use microalgae to produce biofuels [43,44], in the form of H_2_(g) [45–47] or lipids [48–51]. Among all microalgae, *Chlamydomonas reinhardtii* has long been a very attractive model for basic and applied research, due in part to its fast generation time in both liquid and solid media, its suitability for genetic studies due to its two mating types [52–59] and the availability of the sequence of its three genomes (nuclear, chloroplast and mitochondrial), coupled to the possibility to transform each of these genetic compartments [56,60–62]. *Chlamydomonas* is hence very widely used in the study of photosynthesis [55,58,63], chloroplast biogenesis and gene expression [55,58,64] and flagellar assembly and motility [65]. In some instances *Chlamydomonas* has been used as a model organism to study human health-related issues, notably ciliopathies [66–68], as well as for cell factory purposes, such as the production of recombinant proteins [69,70], vaccines [71] or for production of various biochemicals for food, aquaculture, cosmetics and pharmaceutical industries [72,73].

Gaining a better understanding of cell-to-cell heterogeneity in *Chlamydomonas* would thus not only help to tackle fundamental questions, pertaining to population dynamics in this model organism, but will also be of great value to optimize the biotechnological applications it is being used for. In recent years, a few reports have been published on phenotypical cell-to-cell variations in isogenic populations of *Chlamydomonas*, originating from single cells. For example, cell-size and relative cellular starch content were quantified in single cells, leading to the conclusion that, during photosynthesis-driven starch biosynthesis, synchronized *Chlamydomonas* cells possess a wide cell-to-cell diversity both in size and starch content, with the starch-related heterogeneity largely exceeding that of sizes [32]. Rates of starch degradation also largely differed between single cells and, interestingly, were unrelated to the initial cellular starch content [32]. Lipid content and growth-rates were recently studied in *Chlamydomonas* using microfluidics [50] and flow cytometry [74]. The latter study showed a wide distribution of lipid content in both the wild-type and starchless mutant *sta6*, which was not discussed by the authors, but can clearly be seen in the wide distributions of their flow-cytometry results. Vastly diverse lipid contents in single cells of an isogenic *Chlamydomonas* population have recently been demonstrated in a single-cell study using alginate-hydrogel capsules [75]. Finally, two recent microfluidic-based approaches have addressed growth dynamics, *i*.*e*. growth-rates and final yields, for three algal species. When dispersed in individual droplets, *Chlamydomonas reinhardtii*, *Chlorella vulgaris* and *Dunaliella tertiolecta* displayed a wide variety in final algal yield after 10 days, which seemed to depend on the number of initially encapsulated cells (see Discussion section), as well as significant variability in division rates for *Chlamydomonas*, suggesting notable cell-to-cell variations in growth-rate in this species [76]. Comparably, Dewan *et al*. showed that, starting from single *Chlorella vulgaris* cells, final algal yield and division times showed a wide distribution [77].

Apart from the few studies mentioned above, it is as of today poorly known (i) if all cells from a growing culture of isogenic algae exhibit the same growth dynamics and (ii) if *Chlamydomonas* cells age over time, and hence if there is a limit to the number of cell divisions a single cell can undergo in replete, non-limiting, nutritional media. In this study we therefore addressed growth dynamic heterogeneities for individual *Chlamydomonas* populations, originating from single cells, in non-selective and replete liquid media. To this end, we used a millifluidic droplet analyzer, that some of us had originally developed for a diversity scan of bacteria [78]. With key modifications to the previous setup, it is now possible to monitor the growth kinetics of microalgae inside millifluidic drops for 7 days, with the added advantage of conserving their viability and healthy metabolism. Additionally, we developed a sorting and collecting device, which allows sorting and collecting drops of interest at the end of the experiment in 96 well plates, and the use of viable cells contained in these drops for further experiments. Using this newly-developed millifluidic analysis device, we provide direct evidence for cell-to-cell diversity in growth kinetics and cell division capacity of microalgal populations originating from isogenic single cells of *Chlamydomonas reinhardtii*.

## Materials and Methods

### Strains

Three different wild-type strains of the unicellular microalgae *C*. *reinhardtii*, which were stored frozen in liquid nitrogen for the past five years, were used in this study. WT11+ is a descendant of the original 137c strain isolated in 1945 near Amherst (Massachusetts, USA) by G. M. Smith [53,59,79], while WTS24- and WT222+ were generated by multiple backcrosses to WT11+ (see S1 Fig. for a complete genealogy of these strains). The three strains were carefully checked for their wild-type phenotypes in pigment content, photoautotrophic growth, growth in the dark, acetate uptake, fertility and motility. While all checked phenotypes appeared to be “wild-type” for the three strains, they should display multiple genetic polymorphisms due to their genetic history.

### Growth conditions

Strains were grown at 25°C in liquid or solid Tris-Acetate-Phosphate (TAP) media [59,79], containing 100 μg.mL^-1^ of ampicillin to avoid bacterial contamination. Growth was performed under a continuous illumination of 20 μE.m^2^.s^-1^, provided by blue and red LEDs, the spectra of which were Gaussian, with maximal emissions at 460 nm and 635 nm, respectively. Under these dim light conditions, the three wild-type strains do not experience light stress, such as photoinhibition or photooxidative stress, which occur at much higher light intensities [59,63,80]. Cell concentrations of growing liquid bulk cultures were assessed by Malassez-counting under the light microscope. While growing in liquid bulk cultures, cells were diluted twice in the early exponential growth phase (typically 2.10^6^ cells.mL^-1^). They were then synchronized for 4 days under “12h light/12h dark” cycles [79,81,82] and synchrony was verified under the light microscope. At the end of the last dark cycle, cells were again Malassez-counted and the appropriate volume was used for encapsulation and the subsequent millifluidic experiments.

### Samples A and Samples B

**Fig 1:**
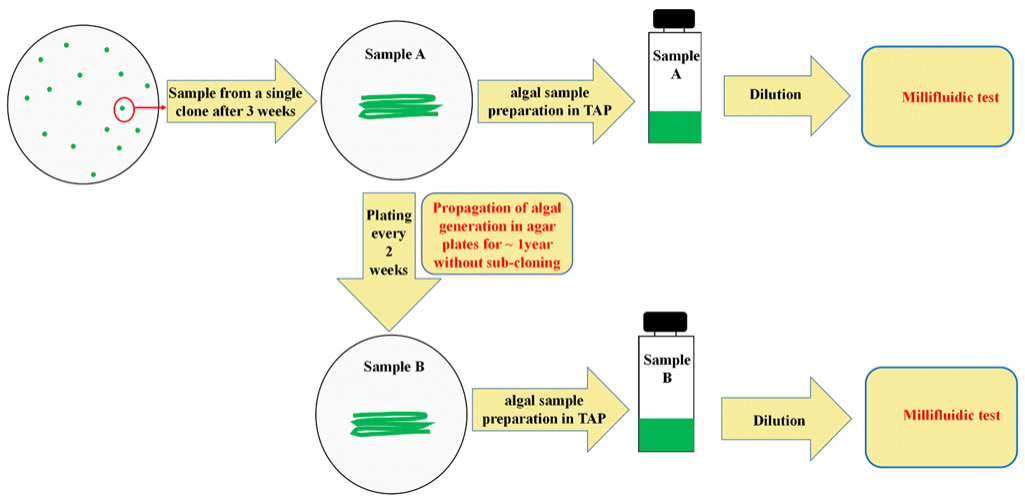
Preparation of Samples A and Samples B of *Chlamydomonas*. Sample A was derived from a single *Chlamydomonas* clone, which was obtained after five consecutive subclonings of the initial strain and is hence highly isogenic by nature. After growing Sample A on agar plates for only three weeks (about 50 generations) it was used for the millifluidic experiments. Sample B was obtained by a series of periodic re-plating of sample A on fresh agar plates every two weeks for about 1 year (about 1000 generations), after which it was used for the millifluidic experiments. For millifluidic experiments, an inoculum from samples A or from samples B on agar plates was transferred to liquid TAP medium. Dilution of algal cultures, when reaching early exponential phase of growth, was repeated twice. After the second round of dilution, liquid cell cultures were synchronized by growing them for four days under 12h light/12h dark cycles. At the end of the last dark cycle, cell synchronization was checked under a light microscope and cells were diluted to the required concentrations with fresh TAP media and immediately used for encapsulation and the start of millifluidic experiments.

Two different batches of each wild-type strain of *C*. *reinhardtii*, named Sample A and Sample B, were used in this study. As shown schematically in Fig. 1. Sample A was obtained after five rounds of subcloning of the initial wild-type strains on TAP-agar plates and hence corresponds to an isogenic, monoclonal, algal population. After the fifth subcloning, algal cells were grown on TAP-agar plates for another three weeks, before being collected as Sample A, using a sterilized pipette tip and transferred to liquid TAP medium for millifluidic experiments.

A fraction of Sample A was streaked onto a new TAP-agar plate, and kept for growth at 25°C under continuous illumination (same conditions as before) for two weeks. It was then transferred to a new TAP-agar plate. Such re-plating was performed every two weeks, for about a year. This corresponds to about 1000 generations, assuming that the average division time on agar plates in these growth conditions is close to the one commonly observed in liquid cultures, *i*.*e*. about 8–9 hours. The resulting sample, named Sample B, was then used for millifluidic experiments in the same experimental setup as for Sample A.

### Millifluidic experiments

**Fig 2:**
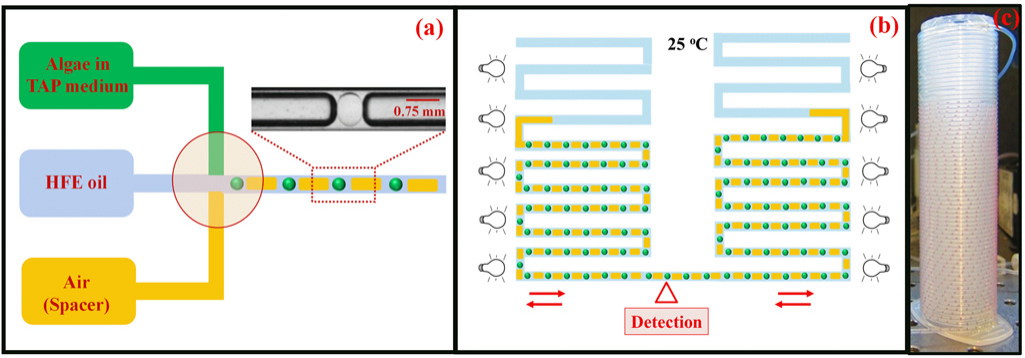
Millifluidic device for the analyses of *Chlamydomonas*. **(a)** Schematic representation of the millifluidic device. Uniform aqueous droplets (green spheres) containing microalgae spatially separated by air spacers (in yellow) dispersed in hydrofluoroether oil (blue) are formed at the cross-junction (red circle). A photograph of an actual millifluidic drop between two air spacers is shown on the upper right corner. **(b)** The transparent FEP tube containing the millifluidic droplets is retained inside an incubator at 25°C, under continuous dim illumination. **(c)** The photograph shows the transparent FEP tube wraped around one of the coils of the millifluidic device. For illustration purposes the drops that normally contain algal cells are filled with a red dye and can bee seen in the lower part of the coil in this picture. The empty part of the tubes can clearly be seen at the top of the coil. When filled with algae, chlorophyll fluorescence emission intensities inside each droplet are measured by the repeated scan of the millifluidic drops during the back-and-forth movement of the millifluidic train, which also ensures appropriate stirring of the encapsulated algae. Fluorescence signals can be converted into cell numbers (see text and S2 and S3 Figs.) and hence growth kinetics can be derived for each algal population. The setup is stable for 140+ hours, allowing growth dynamics to be monitored over a period of about a week.

**Encapsulation**. In the built in-house millifluidic droplet analyzer described in this work, algal cells are encapsulated in mini bioreactors of water-in-oil droplets, having a typical droplet volume of 150 nanoliters. The millifluidic droplet analyzer has two basic parts: the drop-generator and the detector. The drop-generator allows the generation of large numbers of individual millifluidic algal bioreactors, inside transparent Fluorinated Ethylene Propylene (FEP) tubes. The detector module estimates changes in algal cell numbers by measuring the chlorophyll fluorescence intensity inside each drop, using a fluorescence detector. Schematics of the millifluidic droplet analyzer are shown in Fig. 2. In the drop-generator unit (Fig. 2a), a large number of uniform and isolated aqueous droplets containing microalgae, dispersed in a continuous stream of hydrofluoroether (HFE) oil, are generated at a cross junction. To avoid coalescence, nearby algal populations must be spatially separated from each other. For this purpose, a spacer between adjacent millifluidic drops helps the physical and chemical isolation of droplets, thereby eliminating the risk of algal cross-contamination. In a previous millifluidic setup, mineral oil drops were used to separate adjacent bacterial populations [78]. However, millifluidic tests on algae with mineral oil spacer appeared not to be stable beyond 90 hours, due to wetting of the mineral oil and subsequent droplet collapse. Thus, in the present millifluidic setup for microalgae, we implemented air spacers to separate adjacent algal drops. These air spacers, while allowing for the free diffusion of gases to the algae-containing drops, including O_2_ and CO_2_, both of which are important for healthy respiratory and photosynthetic metabolisms of the encapsulated alga, do not allow for the exchange of potentially secreted chemical compounds between individual drops. Hence, each millidroplet is chemically isolated from the others and growth kinetics relate only to the biochemical processes occurring in each individual drop. The droplet train (1100–1200 microbioreactors), containing the growing algal populations separated by the air spacers, is stable for measurement times as long as 140 hrs. To avoid boundary effects, such as high oxygen transfer from the HFE oil to the drops, two large uniform air plugs are generated at both ends of the droplet sequence, to seal the millifluidic train. The droplet sequence thus consists of alternating droplets of algal culture medium and air spacers, retained in transparent FEP tubes having an inner diameter of 0.75 mm. Precise monitoring of droplet sizes after their generation showed that they were extremely uniform, not showing any size variability and hence excluding the possibility that variations in drop sizes/volumes would interfere with our results. S4 Fig. shows three independent biological replicates of typical millifluidic experiments, performed on WT11+ Sample A batches, which illustrate the good reproducibility of results obtained with our setup.

**Detection of fluorescence signals and calibration with regards to cell numbers.** In the detector unit, the cell number is calculated by measuring the chlorophyll fluorescence intensity of algae inside each droplet (Fig. 2b). The excitation light is provided by a blue LED (maximal emission wavelength at 470 nm), which is focused by a 20X-objective on the transparent FEP tube containing the sequence of droplets. The emitted fluorescent light is allowed to pass through a dichroic mirror and an emission filter (maximal emission wavelength at 660 nm) and is finally collected by a photomultiplier tube. Calibration plots of the fluorescence signal, as a function of cell concentration, was obtained for each algal strain by measuring fluorescence intensities of droplets containing known numbers of algal cells (S2a Fig.). Additionally, estimated cell numbers in droplets after millifluidic experiments, as determined by fluorescence measurements and the aforementioned calibration curves, were double-checked by counting the number of cells in the same sorted and collected droplets by flow-cytometry, showing an excellent correlation (S2b Fig.). Finally, in order to check if there were differences between Sample A and Sample B with regards to the correlation between fluorescence signals and cell numbers, these were again checked by flow-cytometry for the three wild-type strains. In each case, Sample A and Sample B showed closely comparable mean fluorescence values with regards to cell numbers (S3a Fig.), as well as very close coefficients of variation of the cell number versus fluorescence (S3b Fig.).

**Growth of algal cells in the millifluidic device.** The growth kinetics of each algal population was monitored by the repeated scan of millifluidic drops, aided by the back-and-forth motion of drops from one coil to the other. The back-and-forth movement of droplets also generates efficient stirring conditions, preventing the aggregation and settling of algal cells, while also ensuring homogenous distributions of dissolved gases (notably O_2_ and CO_2_) inside each drop. To ensure the best possible growth conditions, we sought to recreate the optimal growth conditions used for bulk cultures of *Chlamydomonas* [59,79] in our millifluidic setup. To this end, both the transparent tubes, containing the mini algae bioreactors, and the detector module are maintained inside an incubator kept at a constant temperature of 25°C and the millifluidic drops are exposed to a continuous and uniform dim light during the experiments (20 μE.m^2^.s^-1^, blue and red LEDs, with maximum emissions at 460 and 635 nm, respectively), allowing for healthy mixotrophic growth, while avoiding photo-induced stress or damage, which only occurs at much higher light-intensities [59,63,80].

**Fig 3:**
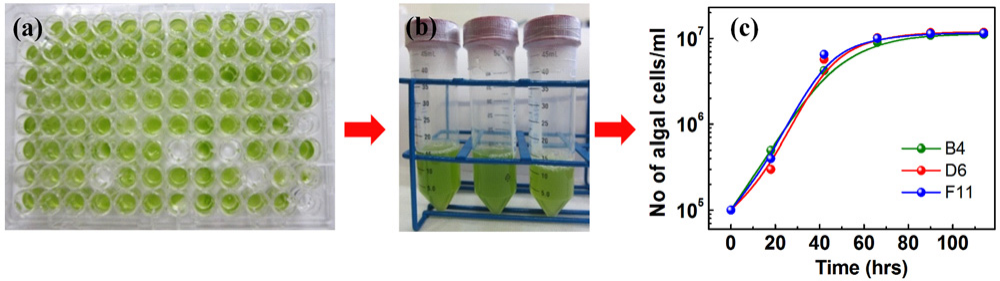
Live and healthy *Chlamydomonas* cells can be sorted and collected after millifluidic experiments for further investigation. **(a)** Millifluidic droplets of fast-growers of Sample A of strain WT11+ were sorted and distributed into a 96 well plate. 200 μL of fresh TAP was added to each well and cells were allowed to grow for a week. **(b)** The photograph shows healthy growth of cells originating from three individual single drops containing fast-growers, collected from the 96 well plate and re-inoculated in new 50 mL bulk cultures. **(c)** Growth curves of the bulk samples prepared from the three sorted and collected droplets of the 96 well plates showing fast and healthy growth.

**Recovery of viable algal cells at the end of the experiment.** One of the challenges in droplet-based screening tests is the recovery of viable cells of interest, belonging to a particular drop, for further studies. To our knowledge, this feat was not achieved in previous studies on microalgae, using either milli- or microfluidic technologies [35,50,76,77,83]. We therefore developed a droplet-sorter, which can collect individual algal drops and either deliver them to a 96-well plate or discard them. After collecting and sorting of the droplets of interest in the 96-well plate, 200 μl of fresh TAP medium were added to each well. The resulting re-suspension could then be used either for plating cells on solid media, or to inoculate new macrocultures for further analysis (Fig. 3). Our setup thereby offers the possibility to select, collect and grow specific algal populations of interest for further analysis, after a millifluidic screening has been performed.

## Results

### Average growth-rates of Chlamydomonas cell populations in millidroplets are comparable to those observed in bulk studies

**Fig 4:**
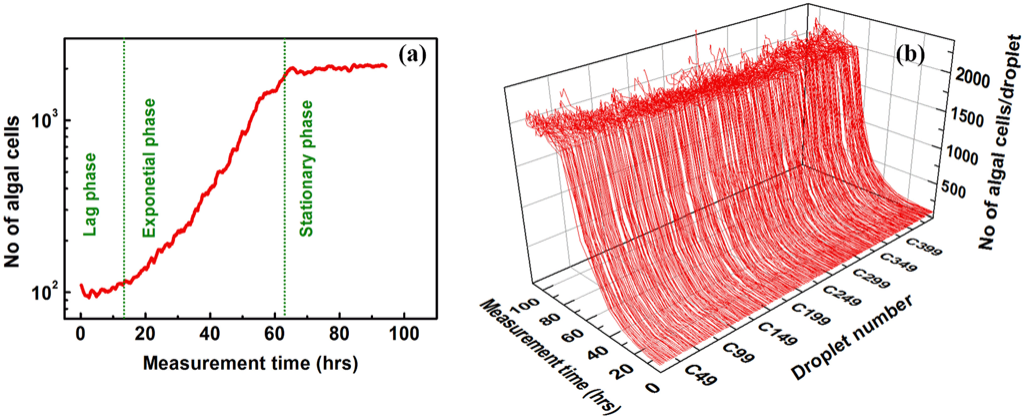
Growth dynamics of *Chlamydomonas* starting from about 100 cells/droplet. **(a)** Growth curve of the WT222+ strain (Sample B) inside one millifluidic droplet, for an initial population size of about 100 cells, showing a lag phase, an exponential phase and a stationary phase, similar to what is commonly observed for bulk cultures. **(b)** Growth curves of 450 millifluidic droplets containing WT222+ (Sample B), from an initial inoculation population size of ~100 cells/droplet, displaying very homogeneous growth dynamics in each drop.

As a first test of the physiological environment provided by our millifluidic droplet analyzer, we measured the growth kinetics of 450 individual algal populations, each starting from an initial inoculate of about 100 cells/droplet in TAP medium, over a time period of 100 hours. Fig. 4a shows a typical growth curve from one randomly selected droplet for sample B of the WT222+ strain. The millifluidic growth curve consists of a lag phase, an exponential phase and a stationary phase, similar to growth curves obtained for samples grown in bulk [59,79]. The doubling time in this experiment was 10–11 hours, which is close to doubling times usually observed in bulk cultures in the same growth conditions (liquid TAP media, dim light) [59,79]. Fig. 4b shows all growth curves, corresponding to each of the 450 millifluidic droplets of the WT222+ strain Sample B. The identical growth curves observed in all individual drops confirms that our growth conditions are homogeneous across all droplets, and over the entire duration of the millifluidic tests. Final algal yield is around 2000 cells in each 150 nL droplet, *i*.*e*. about 1.3 10^7^ cells.mL^-1^, a concentration which matches the ones usually obtained for liquid bulk cultures reaching the stationary phase [59,79]. Thus, growth conditions in each millifluidic bioreactor are a very close replica of a bulk sample, and there are neither nutrient nor other growth limitations inside the millidroplets over the entire duration of our experiments.

### Growth dynamics initiated from single (or very few) isogenic cells of Chlamydomonas disclose the presence of “fast-growers” and “slow-growers”

In an attempt to examine possible cell-to-cell heterogeneity in growth rates, we decreased the size of the initial inoculum within each droplet, down to one, or only a few, cell(s) per drop. To avoid gathering information that would depend on a particular allelic change in a given strain, or on the mating type, we chose clonal/isogenic inoculums, referred to as Sample A (see Material and Methods and Fig. 1) from three different, but closely-related, wild-type strains of both mating types, WT11+ and WT222+ (mating-type +) and WTS24- (mating type-) (see Materials and Methods and S1 Fig.). These three strains are routinely used as *bona fide* “wild-type” strains in laboratory conditions, being highly motile, fertile in crosses and growing vigorously in phototrophic, heterotrophic and mixotrophic conditions. However, the various crosses that were performed to produce each of the three strains (S1 Fig.), as well as the month-to-year period of time during which they were kept by periodic re-plating on TAP-agar medium, before being stored frozen in liquid nitrogen in our laboratory, should have elicited limited genetic polymorphisms through mutations and genetic drift, that may result in changes in growth dynamics between the three strains. Additionally, in order to avoid possible growth heterogeneities in our tests which could be due to encapsulated cells being in varying stages of the cell cycle at the start of the experiment, we first synchronized our bulk cultures for four days, using “12h dark/12h light” cycles [59,79,82]. At the end of the last dark period, synchronization of the cells was checked under the light microscope and synchronized cells were then used for encapsulation. The initial state of the cell cycle for each encapsulated cell in each droplet should therefore be identical and hence not affect the outcome of our experiments.

**Fig 5:**
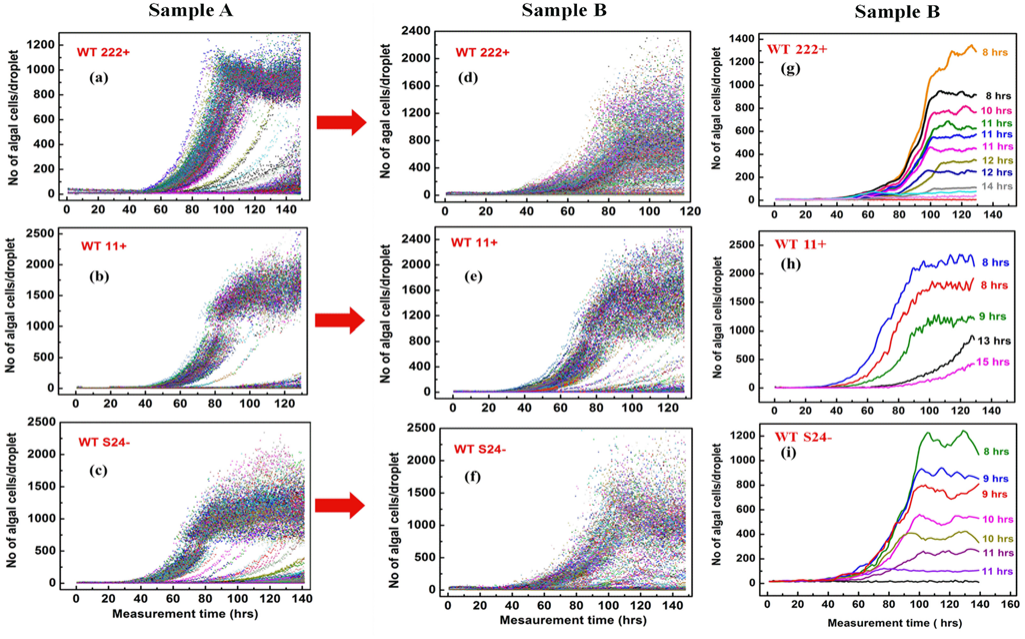
Growth curves of *Chlamydomonas* in droplets containing a low initial cell number (average of 0.16–0.19 cells/drop). Growth curves for Samples A from **(a)** WT222+, **(b)** WT11+ and **(c)** WTS24- or for Samples B from **(d)** WT222+, **(e)** WT11+ and **(f)** WTS24-. Selection of a set of growth curves showing the diversity in growth-rates and final algal yields, observed in Samples B batches for **(g)** WT222+ **(h)** WT11+ and **(i)** WTS24-. Note the sole existence of the two categories of slow- and fast-growers for Sample A batches, and the many intermediate growth phenotypes observed for Sample B batches.

Fig. 5a shows the millifluidic growth curves of sample A of the WT222+ strain, for 0.29 inoculated cells on average per drop, meaning that the train of drops is comprised mainly of a mix of empty drops and drops inoculated with only one cell. It is apparent from the growth curves over 140 hours for Sample A in Fig. 5a, that there are two main distinct algal populations: in 60% of the occupied drops the final algal yield was of 800–1200 cells/droplet, whereas in 30% of the occupied drops, the final yield was below 100 cells/droplet, *i*.*e*. about 6.10^5^ cells.mL^-1^. This latter concentration is typical of *Chlamydomonas* bulk cultures in early exponential phase in common laboratory conditions, and is far from the 1–2.10^7^ cells.mL^-1^ usually reached in the stationary phase [59,79]. Fig. 5b and 5c confirm this main observation, using Sample A from WT11+ and from WTS24-. The corresponding millifluidic growth curves respectively correspond to 0.16 and 0.19 inoculated cells per drop on average. In both cases, two main distinct algal populations were again observed: a majority of drops yield high final cell concentrations (1200–2000), while a minor subpopulation yields a much lower final algal concentration, below 200 cells.

The presence of two populations in these three Sample A batches is mainly due to contrasting generation times. Indeed, while doubling-times range from 7 to 11 hours in drops with a high final algal concentration (>1200 cells per drop), it is much longer (up to 17h) for drops yielding a low final cell number (<200 cells per drop) in the WT222+ and WTS24- strains (Table 1). We could not access the doubling-time of the low-yield droplets for WT11+, because the slow-growing cell populations just entered the exponential phase of growth at the end of the experiment (Table 1). According to the very distinct growth rates of these two populations, which were observed for each wild-type strain, we named these two subpopulations “fast-growers” and “slow-growers”. Because the train of millifluidic drops in our setup is not stable over a period of time longer than 140 hours (due to evaporation of droplets located at both edges of the train), we cannot exclude that drops housing slow-growers would eventually reach the same final algal concentration as fast-growers. We also emphasize that droplets containing fast- or slow-growers were randomly distributed along the millifluidic train, for all three strains, excluding the possibility that the position of the drops in the train might have influenced the growth dynamics of the enclosed cells.

**Table 1:** Division times for the two subpopulations of fast- and slow-growers in Sample A batches from the three wild-type strains.

Sample A	Doubling time for “fast-growers”	Doubling time for “slow-growers”
**WT222+**	7–11 hrs	15–17 hrs
**WT11+**	8–9 hrs	N.D.
**WTS24-**	7–8 hrs	12–14 hrs

Fast-growers typically have a division time ranging from 7 to 11 hours, depending on the strain, while slow-growers have much longer division times ranging from 12h to 17h. Division times for slow-growers of the WT 11+ strain could not be calculated, as they were only at the beginning of their exponential phase (N.D.: not determinable).

To test cell viability at the end of the millifluidic experiments, which, if compromised, would alter the physiological significance of the above observations, we used our newly-developed drop-sorter and collector to harvest drops of fast-growers from Sample A of the WT11+ strain in a 96 well plate (Fig. 3a). After adding 200 μL of fresh TAP media, we re-inoculated bulk macrocultures of 50 mL with three independent fast-growing populations, originating from three independent droplets (Fig. 3b). We then measured growth dynamics of these bulk cultures for an additional 100 hours and observed that all cells were alive, healthy and actively dividing, displaying growth curves identical to what is commonly observed for bulk cultures in the laboratory, with a division time of about 9 hours (Fig. 3c). Our device is therefore capable of sorting and collecting viable cells after a millifluidic experiment, suitable for further analysis. To the best of our knowledge this has not yet been achieved for milli- and microfluidic setups for green algae [32,35,50,76,84], opening a promising way for future uses of the present device, for screening, selecting and collecting *Chlamydomonas* mutants of interest, after random or directed mutagenesis, as well as for selecting subpopulations of interest without mutagenesis.

### When propagated for 1000 generations (Sample B) Chlamydomonas cells display a much wider array of growth phenotypes, including cells with limited division capacities

To understand whether slow-growers would be outcompeted from batch cultures undergoing several hundreds of cell divisions, thus reducing growth heterogeneity in the cell population, Sample A from each strain was allowed to propagate on TAP-agar plates for about 1000 generations (a little more than 1 year) to generate Sample B. We then encapsulated Sample B batches of the three wild-type strains at a low initial inoculum size and monitored their growth kinetics. Fig. 5d shows the millifluidic growth curves for Sample B of WT222+ inoculated at 0.33 cells in average per drops. Cell populations in individual droplets now showed such highly heterogeneous growth kinetics, that it even prevented easy identification of particular subpopulation classes. A highly heterogeneous growth pattern was also observed for Sample B of WTS24- (0.23 cells in average inoculated per drops) (Fig. 5f). In contrast to the very broad, almost continuous, range of growth dynamics observed for the WT222+ and WTS24- strains, Sample B batches of WT11+ (0.17 cells/droplet) (Fig. 5e) showed less heterogeneity, still allowing the distinct observation of the two subpopulations of fast- and slow-growers. The great contrast in behavior of batches from Sample B with respect to batches from Sample A is also illustrated in Fig. 5g-5i, which respectively show millifluidic growth curves in selected drops of Sample B batches of the WT222+, WT11+ and WTS24- strains. In all three cases, cell populations are extremely heterogeneous in final algal yield, with a marked diversity of growth dynamics in individual droplets. While subcategories of fast-growers with a doubling time of 8–10 hours and of slow-growers with longer doubling-times of 12–15 hours can still be identified for all three strains, many intermediate doubling-times were also observed, contrasting markedly with the almost exclusively bimodal distribution of fast- and slow-growers for Sample A batches (Fig. 5a–4c).

**Fig 6:**
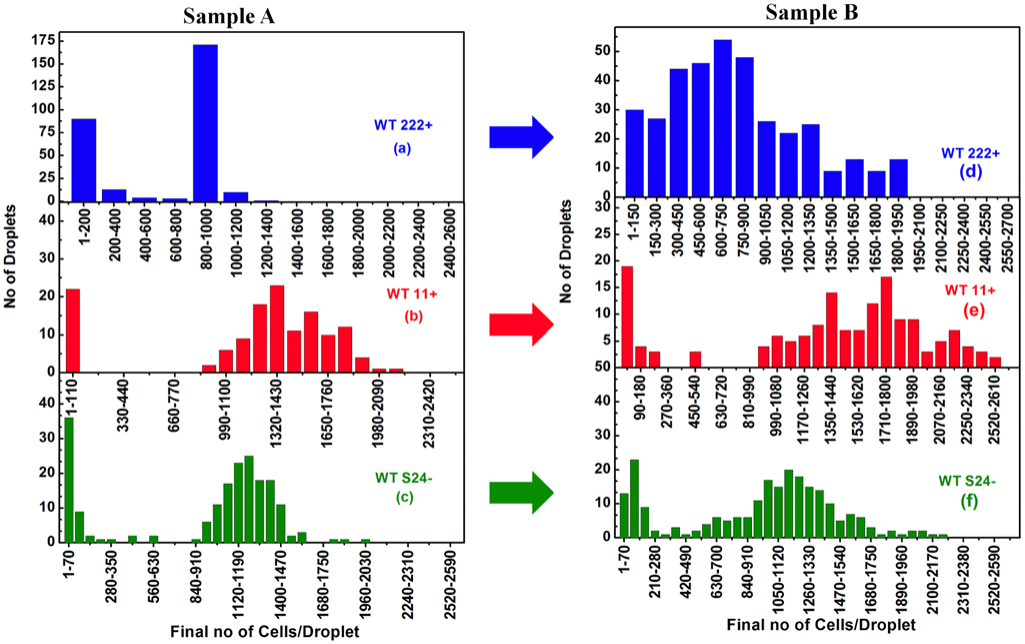
Distributions of final algal yield for samples A and B for each strain. The final algal yield in each drop is shown for Samples A from the wild-type strains **(a)** WT222+, **(b)** WT11+ and **(c)** WTS24-. In each case a population of slow-growers (1–200 for WT222+, 1–110 for WT11+ and 0–70 for WTS24-) and a population of fast-growers (800 and above for WT222+, 990 and above for WT11+ and 1000 and above for WTS24-), is observed. The distributions of final algal yield in samples B is given for the wild-type strains in **(d)** WT222+, **(e)** WT11+ and **(f)** WTS24-. Note the marked broadening of the distribution of final algal yields that still allows identification of two populations of slow- and fast-growers.

The changes in cell-to-cell heterogeneity between Samples A and Samples B are best observed in the statistical comparison of final yield distributions for the three wild-type strains, as shown in Fig. 6. In Samples A (Fig. 6a-6c) the population of slow- and fast-growers are well separated, the slow-growers ranging from 20% (WT11+) to 35% (WT222+) of the cell population in the inoculum. In Sample B, derived from Sample A after propagation for about 1000 generations, the algal yield distribution has much broadened, as shown in Fig. 6d-6f. For WT222+ for example, there is no longer a clear-cut frontier between two subpopulations of slow- and fast-growers (Fig. 6d), whereas this discrimination can still be easily made for Sample B batches of WT11+ and WTS24– (Fig. 6e and 6f). However, even for those two strains, the comparison with the original distribution of their final yields in Sample A (Fig. 6b and 6c), readily shows a significant broadening of their profiles, in between the two initial populations of slow- and fast- growers. Interestingly, in all batches from Sample B, the final algal yield distributions did not follow either a Gaussian or lognormal distribution, but rather a more random pattern. A similar highly multimodal heterogeneity has also been observed in bacterial populations [85]. Our results show that, with time, algal populations acquire a continuous set of growth phenotypes, whose molecular basis requires further investigations.

**Fig 7:**
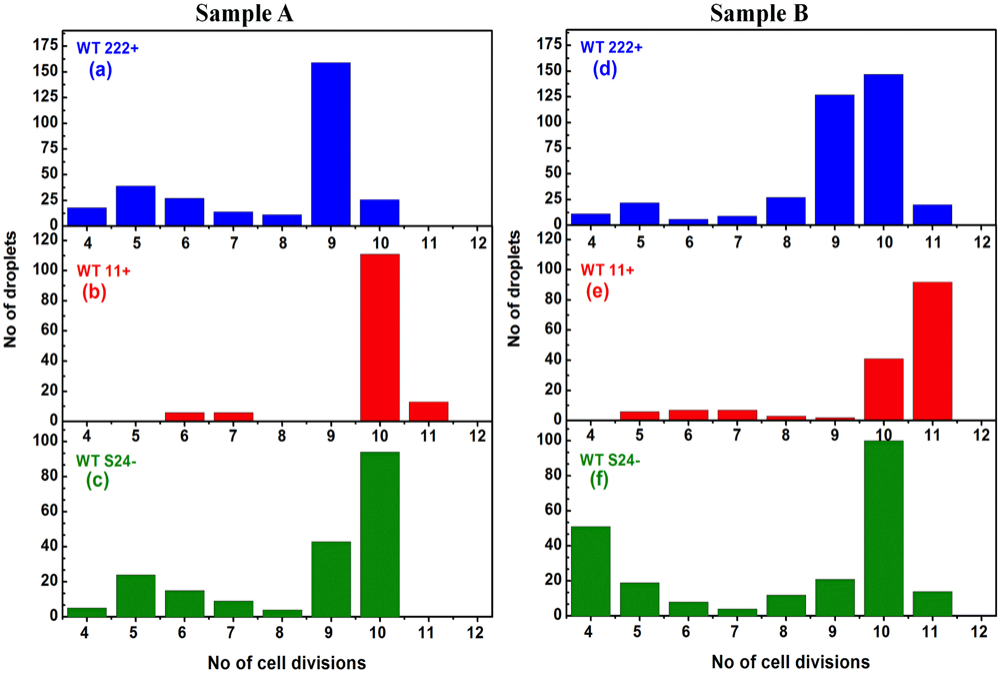
Distribution of cell division numbers among all inoculated droplets of Samples A and B of the three wild-type strains. The number of cell divisions in each droplet was calculated by using the final algal yield and the calculated growth-rate, assuming a single initial encapsulated cell. Results are shown for Sample A (**abc**) and Sample B (**def**) batches for the three wild-type strains WT222+ (**ad**), WT11+ (**be**) and WTS24- (**cf**).

**Fig 8:**
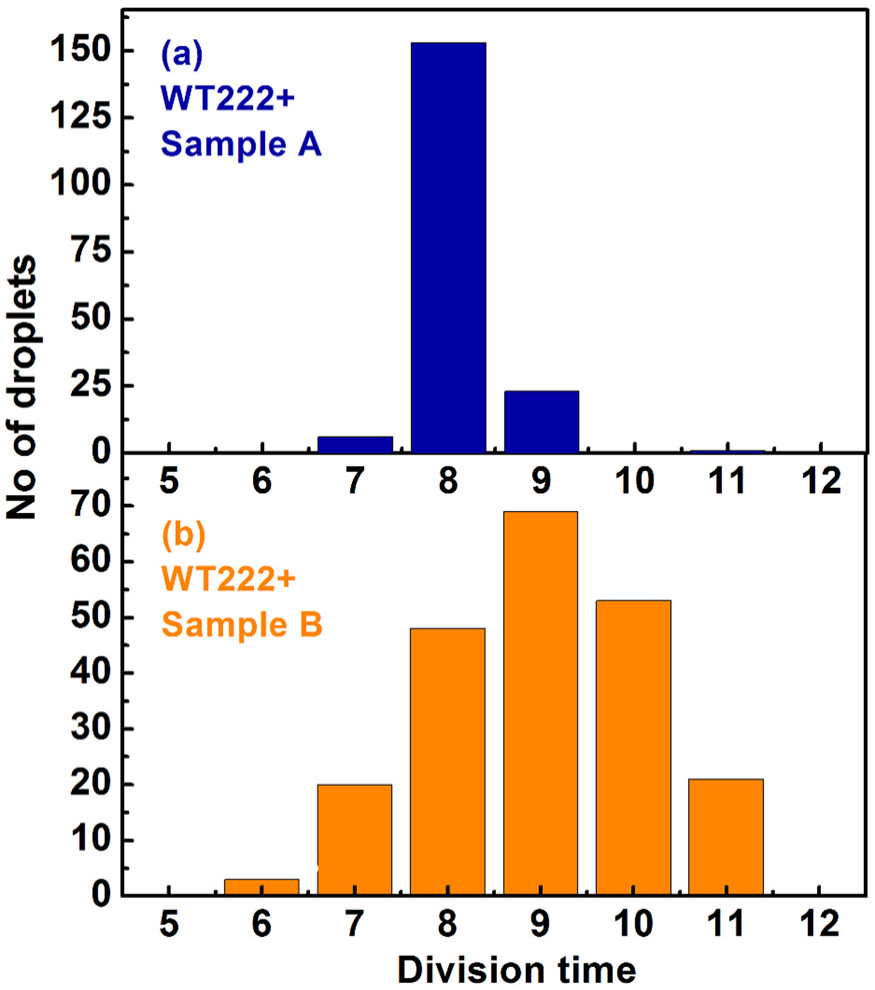
Distribution of doubling times for Sample A and Sample B batches of the WT222+ strain. Only drops with a final algal yield >800 cells are shown, drops lower final algal yields are hence not shown on this graph (see also Fig. 4 and Table 1). A narrow distribution (7–9 hours) is observed for Sample A, whereas a much broader distribution in doubling time is observed for Sample B drops (6–11 hours).

**Fig 9:**
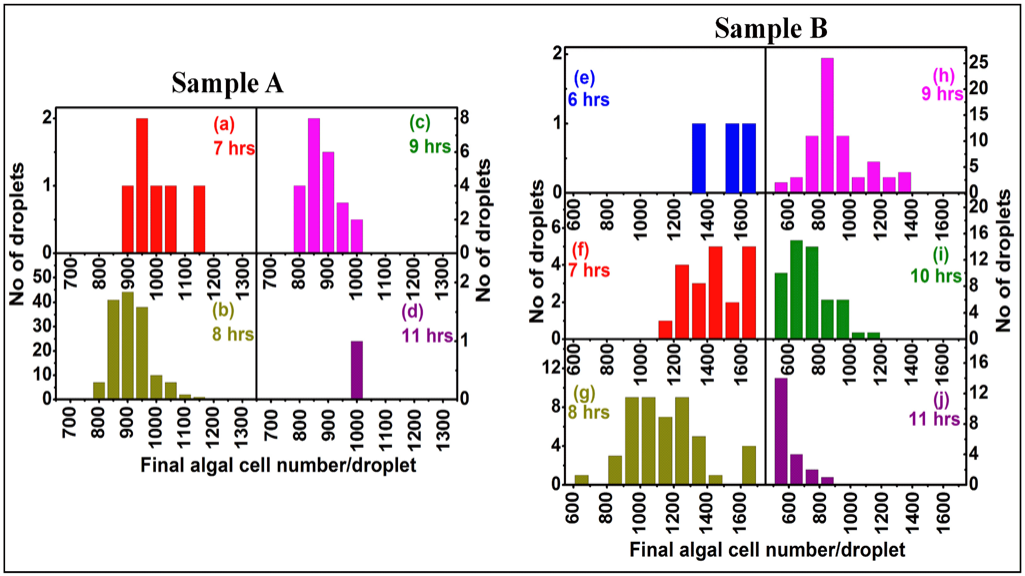
Final algal yield for droplets with similar growth-rates for Sample A and B batches of WT 222+. The final algal yield (x-axis) is shown for droplets (y-axis) having the same (or very close) division time (7 to 11 hours) for Sample A (**abcd**) and Sample B (**efghij**) batches of the WT222+ strain. Drops containing cells dividing with the same growth-rate can give rise to different final algal yields, suggesting that in individual droplets growth arrest has occurred after a varying number of cell-divisions, independently of doubling-time. The observed diversity is more pronounced for Sample B batches (right panel) with regards to Sample A batches (left panel).

Ultimately, differences in final algal yield reflect differences in the number of cell divisions that have occurred in individual droplets. The number of divisions was calculated using the growth-rate in each individual droplet and the final algal yield, assuming a single-cell encapsulation at the beginning of the experiment. Fig. 7 shows that there is indeed a large diversity in cell-division numbers between individual drops, both for Sample A (Fig. 7a-7c) and Sample B (Fig. 7d-7f). It varied widely, from as low as 4 to as high as 11, over the course of the experiment (Fig. 7), with batches from Sample B showing a slightly larger variety in cell division numbers than in Sample A. That the number of cell-divisions varied between individual droplets could originate from two distinct phenomena: (i) a heterogeneity in doubling-time between individual populations and/or (ii) a heterogeneity in the number of cell divisions that have occurred in different droplets but *without* a change in doubling-time. As has been observed already for Sample A, there is indeed a heterogeneity in doubling-times, resulting in slow-growers and fast-growers (7–11 hours and 15–17 hours, respectively), with a clear diversity even inside these two categories (see Fig. 5a-5c and Table 1). Interestingly, there is a broader distribution in doubling-times for Sample B batches as is illustrated in Fig. 8 for the WT222+ strain, for drops with a final algal yield >800 cells (the very low-yield, long division time droplets are not included in this figure). While doubling-times show a narrow distribution peaking mainly at 8 hours, with minor populations at 7 and 9 hours for Sample A batches (Fig. 8a), batches of Sample B of the same strain show a much wider distribution, ranging from 6 to 11 hours (Fig. 8b). It is of note that droplets containing algae dividing with a similar doubling-time do not necessarily end up with a similar final algal yield. This is readily observed in Fig. 5g-5i, showing growth curves in a few drops from the three wild type strains, where division times are indicated next to the plateaus corresponding to the final cell concentrations. This phenomena is best seen in Fig. 9, where the distribution of final algal yields are compared among cell populations with similar doubling times, ranging from 6 to 11 hours for the WT222+ strain. With Sample A, drops with uniform doubling-times, ranging from 7 to 9 hours, give rise to droplets with final algal yields ranging from 800 to 1100 cells/drop (Fig. 9a-9c). With Sample B, the variety of final algal yield for droplets with the same doubling-time is even more pronounced. For example, drops with doubling times of 8 hours (Fig. 9g) and 9 hours (Fig. 9h) display final algal yields extending in the range of 600–1600 and 500–1400 cells/drop respectively (Fig. 9g and 9h). These results suggest that cells inoculated in different drops do not have the same ability to perform similar numbers of cell divisions, even though they have the same generation time. This in return suggests that an arrest in cell division develops earlier in some drops than in others, regardless of the doubling-time. Here again drops with identical behaviors were randomly scattered throughout the millifluidic train, excluding the contribution of a position effect to our observations.

### Re-encapsulation of cells derived from a single droplet with a given growth dynamic, regenerates a larger diversity in growth dynamics

**Fig 10:**
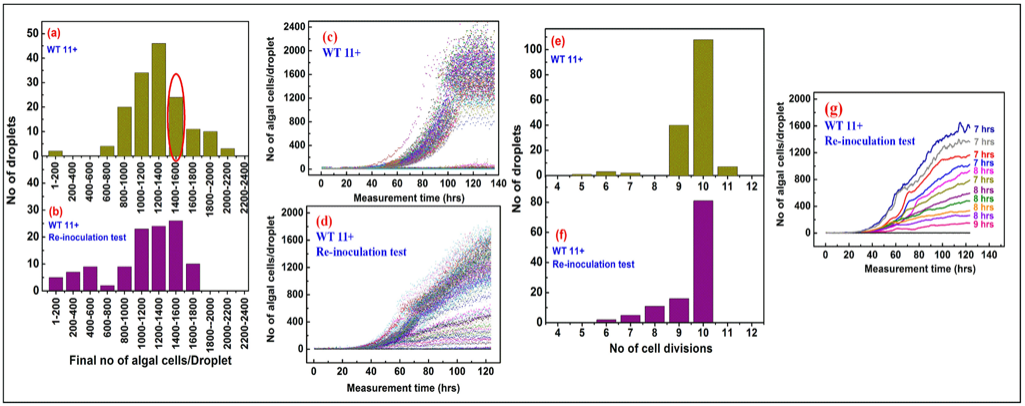
Re-inoculation of a recovered single millifluidic droplet of the Sample A WT11+ strain yields a new algal population displaying increased growth-parameters diversity. **(a)** Statistical distributions of final algal yields in millifluidic drops for freshly subcloned (Sample A) *Chlamydomonas* wild-type strain WT11+. One drop, originating from fast-growers from the 1400–1600 category (circled in red), was sorted and collected at the end of a millifluidic experiment (*i*.*e*. after having grown for 10–11 generations). **(b)** 200 μL of fresh TAP was added to the drop and the cells were then re-encapsulated for a new millifluidic growth experiment at an occupancy of 0.12 cells/drop. A larger diversity of growth phenotypes is observed for the re-inoculated population when compared to the monoclonal original population, as can be seen by comparing the distribution of final algal yields (compare **a** and **b**). A larger variety of growth dynamics is also observed for the re-inoculated sample, when comparing their millifluidic growth curves (compare **c** and **d**).

The increased heterogeneity in growth dynamics for Sample B of the three wild-type strains, should be due to their propagation on agar plates for 1000 generations. This propagation may lead to the accumulation of spontaneous mutations over time, which would affect growth dynamics of individual mutants (see Discussion section). Therefore we performed an additional millifluidic experiment with Sample A from the WT11+ strain, with a low initial drop occupancy of 0.12 cells per drop (Fig. 10). The behaviour of these Sample A batches of WT11+ (Fig. 10a, 10c and 10e) was nearly identical to the behaviour observed for Sample A batches in previous experiments (Fig. 5b, 5e and 5h), including the clearly-defined subpopulations of slow- and fast-growers, with little to no intermediate growth dynamics (Fig. 10c and 10e). At the end of the millifluidic experiment, we selected one particular drop, containing a population of fast-growers (about 1600 cells as final algal yield, circled in red in Fig. 10a). These cells still should be isogenic, because they originate from a single cell from isogenic Sample A and have been propagated in non-selective conditions for only 10–11 generations (about 100 hours of active growth, Fig. 10c), a much too short period of time for the population to be subjected to significant genetic variations. We recovered the cell content from this drop and used it for re-encapsulation, at a low initial occupancy (0.12 cells per drop). The subsequent analysis of the growth-dynamics in that experiment (Fig. 10b and 10d) disclosed an unexpected heterogeneity in this cell population, recovered originally from a single drop of fast-growers. The final algal yield had a broad distribution with two peaks at about 500 and 1300 cells per drop (Fig. 10b), and the slow-growers had now a much wider distribution than originally observed for Sample A (Fig. 10a). Whereas the numbers of cell divisions in individual droplets showed significant but limited variability (Fig. 10f), the much wider distribution in final algal yields (Fig. 10b) rather originated from a pronounced variety of doubling-times between individual droplets (Fig. 10g). This re-encapsulation experiment thus shows that growing an isogenic population for only 10–11 generations in liquid media is sufficient to generate a wider diversity of growth phenotypes than what was observed for the initial Sample A (Fig. 5a-5c and Fig. 10a and 10c).

### A further decrease in inoculum size does not change the observed heterogeneities in growth dynamics

**Fig 11:**
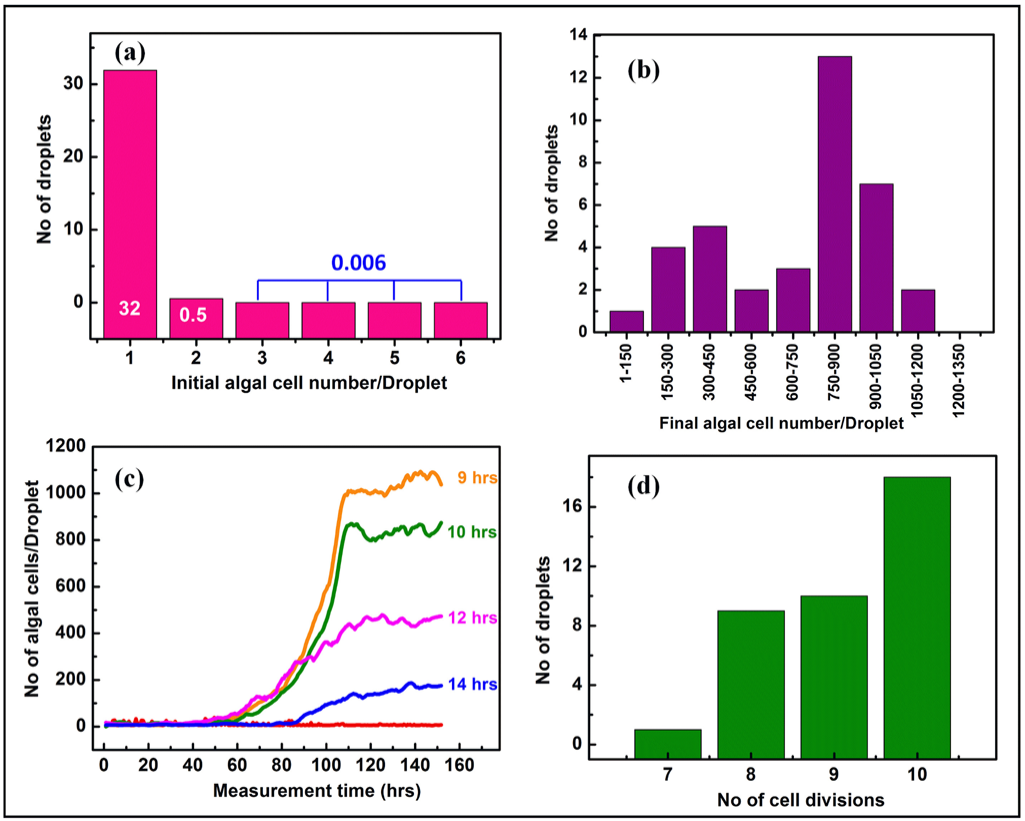
Growth kinetics for *Chlamydomonas* cultures in millidroplets inoculated with no more than one cell. **(a)** Poisson distribution showing the number of droplets that should encapsulate single (32), two (0.5) or more than two (0.06) algal cells, for an overall droplet occupancy of 0.07. Multiple initial occupancy of droplets is hence highly unlikely and growth kinetics relate to the growth dynamics for populations originating from a single cell. **(b)** Distribution of final algal yield for Sample B from WT222+, showing a wide distribution of growth phenotypes. **(c)** Millifluidic growth curves for single cell encapsulations of sample B from WT222+, showing a wide variety in both final algal yield and division time. **(d)** Number of cell divisions in each drop for WT222+ (Sample B) for the single-cell encapsulation experiment.

In all the experiments described above, the inoculum size ranged from 0.12 to 0.33 cells/drop, which was close to, but did not fully, eliminate the chances of having a few droplets entrapping more than one cell at the start of the experiment. According to a Poisson distribution, only 5–15% of all droplets could have encapsulated two or more cells at the beginning of the experiment. We refrained from further decreasing the initial drop occupancy in the previous experiments, in order to keep a large enough number of algae-containing drops in the millidroplet train, to preserve the statistical significance of our results. However, to fully rule out a contribution of limited variations in initial droplet occupancy to the observed heterogeneity in growth dynamics, we carried out a millifluidic test with sample B from WT222+ at the very low droplet occupancy of only 0.07 inoculated cells per drop. Fig. 11a shows that, according to a Poisson distribution with this occupancy, 32 droplets should encapsulate single cells, while only 0.5 drops would encapsulate two algal cell, and 0.06 drops would have more than 2 initial cells, along with 935 empty droplets along the millifluidic train. Populations derived from this single-cell-encapsulation experiment still showed large heterogeneities in cell growth dynamics (Fig. 11b-11d). A large diversity in doubling-times (Fig. 11c) and in final algal yield (Fig. 11b) is readily observed. Fig. 11c displays a set of growth curves leading to different final algal yields, with contrasting generation times, ranging from 9h to 14h. Fig. 11d shows the distribution of cell-divisions per droplet, ranging from 7 to 10. Thus the contents of a single droplets from this experiment, solely derived from single-cell-encapsulations, still show large heterogeneity in cell growth dynamics.

## Discussion

### Development of a reliable millifluidic device preserving Chlamydomonas physiology and thus allowing the study of its growth dynamics

Studying single cell behaviors of an isogenic population of *Chlamydomonas reinhardtii* requires a set up that preserves growth dynamics similar to those usually found in batch cultures, while allowing the safe recovery at the end of the experiment of a clonal population of viable cells, amenable to further molecular analyses. With generation times of fast-growers reproducibly ranging from 7 to 10 hours, with an initial occupancy ranging from as low as 1 to as high as 100 cells per droplet, the present millifluidic device qualifies for providing the physiological environment required for optimal photoheterotrophic growth of *Chlamydomonas*. Furthermore, cells in individual droplets remain viable at the end of the experiment and can be recovered by our newly-developed sorting and collecting device, for re-inoculation of new macrocultures or for plating on solid media.

With this set up, we observed heterogeneities in growth dynamics of populations originating from single cells, which required a close examination of possible artefacts, before any attempt to assess the physiological significance of these observations. The widespread distribution of both final algal yields and growth-rates was not the result of a heterogeneity in the distribution of initial numbers of encapsulated cells, contrary to what was reported in other microfluidic studies. For instance, Pan *et al*. observed a positive correlation for *Chlamydomonas* [76] and Dewan *et al*. a negative correlation for *Chlorella* [77]. With our setup, droplet occupancy in most of our experiments ranged from 0.12 to 0.33 cells per drop, which corresponds to Poisson distributions giving a maximum range of 5–15% of total drops with an initial occupancy higher than one. However, the highest occupancies of 0.29 and 0.33, corresponding to experiments with WT222+, did not show more contrasted growth heterogeneities between droplet populations, when compared to lower occupancies, ranging from 0.12 to 0.19, for strains WT11+ and WTS24^-^. In addition, experiments performed with a very low initial occupancy of 0.07, which rule out the statistical probability of encapsulating more than one cell at the beginning of the experiment, still showed a wide variety of growth dynamics in individual drops.

We also used synchronized seed cultures before the millifluidic experiments, in order to exclude a possible heterogeneity in growth dynamics that would merely originate from differences in the stage of the cell cycle at which the millifluidic experiment had started. If there would be some contribution of a limited asynchrony, that indeed is observed with synchronized cell cultures of Chlamydomonas [82] it would likely be ascribed to stochastic variations in the duration of particular stages of the cell cycle, that are part of those phenomena that the present study aims at describing. Finally, the observed heterogeneities were not dependent on the position of the droplets in the millifluidic train, as demonstrated by their random distribution along the train of drops. Since we observed growth heterogeneity with clonal populations from three distinct wild-type strains, two of mating type plus and one of mating type minus, all derived from strain 137c but possibly with multiple polymorphisms, we are confident that our observations reflect a genuine behavior of *Chlamydomonas reinhardtii*. We hence conclude that there is considerable and reproducible cell-to-cell heterogeneity in isogenic *Chlamydomonas* populations with regards to growth parameters.

### Stochastic variations likely govern growth dynamics of isogenic populations of Chlamydomonas

A most striking observation is that Sample A, which corresponds to isogenic populations of recently subcloned cells of *Chlamydomonas*, yields two subpopulations of fast- and slow-growers, with contrasted lag phases before resuming growth and division times that differ by about a factor of two. The same heterogeneity was observed for the three strains we used in our study, yielding a population of 20–35% of the droplets that contain less than 200 cells after 140 hours of growth (slow-growers), when in 65–80% of the droplets, the other population accumulates about 1000–1500 cells during the same laps of time (fast-growers). These two original populations generated by clonal cells, as observed in Samples A, were still observed in Samples B, when these strains were propagated on solid media for 1000 generations before performing the millifluidic experiment, even though the heterogeneity in growth dynamics was now considerably increased. Heterogeneities in growth-rates and/or cell division number in unicellular organisms have been reported earlier in the literature. It was shown, as soon as 1932, that individual bacteria of an isogenic population display considerable diversity in their growth-rate, with slow- and fast-growing bacteria, as well as many intermediate growth phenotypes [1]. Since then, the existence of slow-growers and persisters in isogenic bacterial populations has become a well-known and extensively studied phenomena. Bacterial persistence enables a cell population to resist antibiotics, which often target actively dividing cells [86]. Persistence has been shown to result from stochastic induction of toxin-antitoxin systems, regulated by (p)ppgpp, and a recent study nicely demonstrated that an exponentially growing population of *E*. *coli* produces rare cells that stochastically switch into persistent slow-growth mode, even in the absence of antibiotics. These slow-growers are indeed resistant to antibiotics and are fully able to resume active growth [87]. Usually bacterial persisters are detected in very small proportion of 10^-4^–10^-6^ [86,87]. Even when considering *hipA7* mutants of *E*. *coli* that can generate persisters at much higher frequencies of 10^-2^ to 10^-3^ [88,89], it must be noted that this proportions are orders of magnitude lower than the proportion of slow-growers that we detected in *Chlamydomonas* populations. Recent studies of unicellular *Salmonella* cells showed great cell-to-cell variability in their division parameters, when they are located inside the cytosol of an eukaryotic host cell: although isogenic and placed in a very tightly homeostatic environment, some cells divide actively, whereas others are in a dormant, non-dividing state [90–92], which is reminiscent of slow- and fast-growing phenotypes.

The existence of fast- and slow-growers in this eukaryotic microalga is consistent with earlier observations reporting contrasted division abilities between cells from isogenic and synchronized cultures of *Chlamydomonas* [82,93]. Indeed, we noted that re-encapsulation of fast growers from a single drop of an isogenic population (Sample A) again generated a diversity of growth dynamics. This behavior strongly suggests that some stochastic variation governs the growth dynamics of populations originating from single cells. Similar variations were reported recently for starch degradation in *Chlamydomonas* [32], which also showed high cell-to-cell heterogeneity, but were unrelated to the initial cellular starch content. A similar stochastic variation was observed for aging *E*. *coli* cells: “old” cells can still give rise to rejuvenated offspring, with fast growth and high division rates [94,95].

Cell-to-cell variability in cell-fate decision has also been demonstrated in single isogenic yeast cells [16]. In this study, the authors studied the response of single cells to pheromones, that normally trigger a transition from vegetative growth to the initiation of mating events, including induction of gene transcription, cell-cycle arrest and changes in morphology, through the activation of known signalling pathway (involving G-coupled receptors and MAP kinases). Using reporter genes, they showed that individual cells react with significantly different amplitudes to the pheromone treatment and that cell-fate decisions were not homogenous among the population. Cell-to-cell variation was shown not only to be caused by random fluctuations in gene transcription and translation, *e*.*g*. genetic expression “noise”, but phenotypic variety was also generated by large differences in the capacity of individual cells to transmit signals through signalling pathways [16]. This example nicely illustrates how cell-fate decisions in yeast relating to growth dynamics (divide, not divide, grow, stop to grow) can be stochastically different between isogenic cells.

It is also worth considering possible epigenetic variations that could play a major role in adaptive strategies to diverse environmental conditions and their dynamics (reviewed in [96] and [97]). Although epigenetic regulation of gene expression is well-studied in other organisms, it is only in its infancy in *Chlamydomonas*. It has recently been shown that chromatin remodeling does occur in *C*. *reinhardtii* and plays an important role in gene expression regulation [98,99]. It is hence possible that the wide variety of growth phenotypes that we observe in our study is related to differing states of the chromatin in individual cells at the encapsulation step, which might affect the expression of genes regulating cell-growth and cell-division, leading to populations displaying varying growth parameters.

### Spontaneous mutations alone are unlikely to explain the wide array of growth phenotypes of a propagated cell population of Chlamydomonas

The increased cell-to-cell heterogeneity in both growth rate and cell division capability, that we observed in Sample B, where the cell population results from about 1000 rounds of divisions from an originally isogenic population (Sample A), may suggest a contribution of spontaneous mutations and genetic drift during this prolonged time of propagation in our laboratory conditions. In a recent report [100], multiple strains of *C*. *reinhardtii* were maintained for approximately 1000 generations on solid media (same setup as our Sample B) under relaxed selection, by transferring a single cell every 10 generations to new growth medium. The authors showed that the mean fitness of the strains, as defined by maximum growth parameters in liquid medium, tended to decline with generations. They suggested that this phenotypic drift should be ascribed to the sole occurrence of spontaneous mutations over the course of the experiment. Here we noted an enrichment in droplets housing the highest final growth yields in Samples B as compared to Sample A, which suggests a genetic drift in the opposite direction that could result from our replating procedure—every two weeks—that may enrich our batches in fast growing cells. However, that our observed growth heterogeneities are solely due to the accumulation of genetic variants is unlikely, based on three recent and independent evaluations of the mutation rate/base/generation in *C*. *reinhardtii* [101–103]. In one study [102], where *Chlamydomonas* cells were grown under continuous light in TAP medium (conditions very similar to our setup), with 283 serial dilution of exponentially growing cells with fresh medium (about 1880 generations), the authors estimated the mutation rate in *Chlamydomonas* to be around 1.5×10^-8^/base/generation, representing about 1.7 mutations/generation given the 111.1 Mb size of the *Chlamydomonas* nuclear genome. In another study where *Chlamydomonas* was propagated for 350 generations, the spontaneous mutation rate by whole-genome sequencing of a strain was estimated at a much lower number of about 3.2×10^-10^ mutations/base/generation, *i*.*e*. 0.04 mutations/generation [101]. A third study obtained by whole-genome sequencing after 1730 generations has evaluated the spontaneous mutation rate in *Chlamydomonas* at an even ten times lower rate of 3.7×10^-11^/base/generation, *i*.*e*. about 0.004 mutations per generation [103]. Our Samples B that were propagated for 1000 generations would hence harbor from 0.3 [103] to 128 [102] mutations leading to changes in protein sequences, assuming that 7.7% of all mutations lead to amino-acid substitutions as described in [102]. Even when taking into account the highest value from Perrineau et al. [102], the number of mutations found in the population is far too low to explain by its own the extreme diversity of growth phenotypes seen in almost all the droplets of Samples B and the wide (almost continuous) distribution of division-times from as low as 6 to as high as 20 hours in three different strains.

### Different cell division capabilities after thousand rounds of cell division (Sample B) suggests an, as yet unknown, aging process in Chlamydomonas

As we discussed above, stochastic switches should contribute to growth heterogeneity among Chlamydomonas cells. However, we observed that the heterogeneity in the number of divisions that undergo cells displaying the same (or very similar) generation times is mostly observed when they have been kept on agar plates for about 1000 generations before recording the millifluidic experiment. It was observed to a lesser extent with cells that have been propagated for only 10–11 generations prior to a re-encapsulation experiment, and it was absent from clonal cells in Sample A. Although phenotypic heterogeneity due to stochastic switches may increase with the number of cell divisions, the above observations rather suggest that Sample B is enriched in aging cells that are not able of undergoing the same number of mitotic divisions as younger cells. However, *Chlamydomonas* is reported to divide symmetrically, with usually four identical daughter cells being liberated in the medium after two rounds of division [54,79]. There are, as of yet, no reports of any significant cytological or molecular differences between daughter cells in *Chlamydomonas* that would serve as a basis for an aging process. However, while *E*. *coli* can be kept alive for an apparent unlimited number of divisions in unstressed and replete media [104], it was recently shown that, following repeated cycles of reproduction through automated time-lapse microscopy, *E*. *coli* actually possess an old and a new pole during its life cycle and division. The daughter cells inheriting the old pole of the mother cell exhibit diminished growth rates, decreased offspring production, and an increased incidence of death [94]. The authors concluded that the two supposedly identical cells produced during cell division are functionally asymmetric, with cells originating from the old pole being an “aging” parent, still capable of repeatedly producing rejuvenated offspring, but ultimately doomed to growth arrest and even cell death. Although these results seemed conflicting with a follow-up study by Wang *et al*. [105], further investigations clearly showed that aging and rejuvenation occurred simultaneously in a population and that, indeed, cells derived from the old pole showed increasingly longer doubling times [95]. New experimental and theoretical methods have recently been developed to address these questions further [106].

Aging in unicellular eukaryotes have been reported, for instance with *S*. *pombe*, *C*. *albicans*, *K*. *lactis*, and *C*. *neoformans*, some of which perform symetrical divisions by fission, similar to what is observed in *Chlamydomonas* (reviewed in [23,24,107]). Their study has greatly benefited from the knowledge of the aging process in the well-studied, asymmetrically dividing, budding yeast *Saccharomyces cerevisiae*, that has been reported as early as 1959 [17]. Replicative aging, chronological aging and uneven distribution of cellular components between the two daughter cells, most notably accompanying asymmetric cell division, all contribute to the generation of an “older” and a “younger” daughter cell, the older one ultimately stopping to divide [23–25]. These studies led to the discovery of longevity genes, which, interestingly, often proved to be conserved in multicellular eukaryotes. Many studies document telomere shortening, as a contributing cause to aging and senescence in eukaryotic cells [24,107–118]. While *Chlamydomonas* has linear nuclear chromosomes [52,60], which are bordered by telomeric sequences (TTTTAGGG), similar (but not identical) to those found in higher plants [119–123], it has not been shown, up to now, how these telomeres are maintained and how they might shorten over time, impacting the lifespan of the organism. Aging could also be triggered in *Chlamydomonas* by uneven transmission in the number of copies of the chloroplast chromosome, as suggested in [124]. In a regular mitotic cell division, the polyploid chloroplast genome (around 60–100 copies) is thought to segregate in roughly equal amounts to the daughter cells after cell-division. Using the insertional mutant *moc*, which is defective in equal splitting of chloroplast genomes during cell-division, it was shown that, when daughter cells only inherited a low number (2–4 copies) of the chloroplast genome, they degenerated and stopped growth more quickly than daughter cells containing a higher genome copy number [124]. Low-copy cells divided slightly slower and only reached 1/3 of final algal yield when compared to high-copy cells. Low-copy cells also aged more rapidly, as defined by loss of pigmentation and division capacity, more rapidly than high-copy cells.

## Conclusion

The present study, with the ability to recover viable cells at the end of the millifluidic experiment, offers a unique opportunity to further study, notably at the molecular level, cell-to-cell variability in the model organism *Chlamydomonas*. Such variability certainly reflects cell-to-cell heterogeneities triggered by stochastic switch-like events which are, as of yet, very poorly documented in *Chlamydomonas*, when compared to the vastly expanding literature on phenotypic cell-to-cell heterogeneities in other organisms. It may also arise from an as yet undescribed aging mechanism in *Chlamydomonas*. Finally, the availability of the present millifluidic device, which enables the recovery, sorting and collecting of viable cells at the end of the experiment, should also prove very helpful in future studies aimed at identifying subpopulations of *Chlamydomonas* displaying a phenotypic trait of interest, for biotechnological applications or basic research.

## Supporting Information

S1 Fig Genealogy of the three wild-type strains of *C*. *reinhardtii* used in this study. Strains used in this study are shaded in blue. WT11+ and WT12-, used in the authors laboratories, are descendants of the original 137c strain isolated in 1945 [53,79]. Crosses between these two strains yielded WTN+ (in brown) and WT69-, which was backcrossed twice to WT11+, firstly yielding WT4- and secondly yielding WTA1- and WTB1+. A cross between these strains yielded WTS3- (in orange) which was again backcrossed to WT11+, yielding strain WTS24-. Finally, this strain was backcrossed to the first generation descendant WTN+ to yield WT222+. The three strains WT11+, WTS24- and WT222+ are thus closely related but should have accumulated multiple genetic polymorphisms due to these crosses and to genetic drift through the overall elapsed time of their independent replating.
(TIF)

S2 Fig Calibration experiments relating fluorescence signals to cell count for each strain from Samples A. **(a)** Calibration curves showing the fluorescence signal (y-axis, arbitrary units) as a function of known numbers of algal cells inside droplets (prepared from solutions of known concentration) for Samples A from three different wild-type strains WT11+, WT222+ and WTS24-. **(b)** Three droplets originating from a millifluidic growth experiment of *Chlamydomonas* were assessed for final algal cell count either by fluorescence measurement using calibration curves shown in **(a)**, as well as by directly counting the cells through a flow cytometer. A good correlation is observed between the two quantification methods.
(TIF)

S3 Fig Reliability of cell counts by fluorescence measurements between Samples A and Samples B. For the three different wild-type strains WT11+, WT222+ and WTS24-, the relationship between fluorescence and cell count was established by analyzing solutions of known algal concentration using a flow cytometer. **(a)** The intensity of each distribution is represented by the position of the center of the distribution (mean-X) for both samples A and samples B. **(b)** The cytometer fluorescence measurements of Samples A and B of the three wild-type strains showed very similar coefficients of variation (CV-X%) confirming that the variability in chlorophyll content of cells in Sample A and B are identical **(b)**.
(TIF)

S4 Fig Reproducibility of millifluidic experiments. Comparison of the distributions of final algal yields from Sample A batches (WT11+) for three independent millifluidic experiments, showing a good reproducibility of millifluidic experiments.
(TIF)
