# Supplementary figures and images for: A Millifluidic Study of Cell-to-Cell Heterogeneity in Growth-Rate and Cell-Division Capability in Populations of Isogenic Cells of Chlamydomonas reinhardtii
Source: PLoS One. 2015 Mar 11;10(3):e0118987. doi: 10.1371/journal.pone.0118987 (PMC4356620; doi:10.1371/journal.pone.0118987)

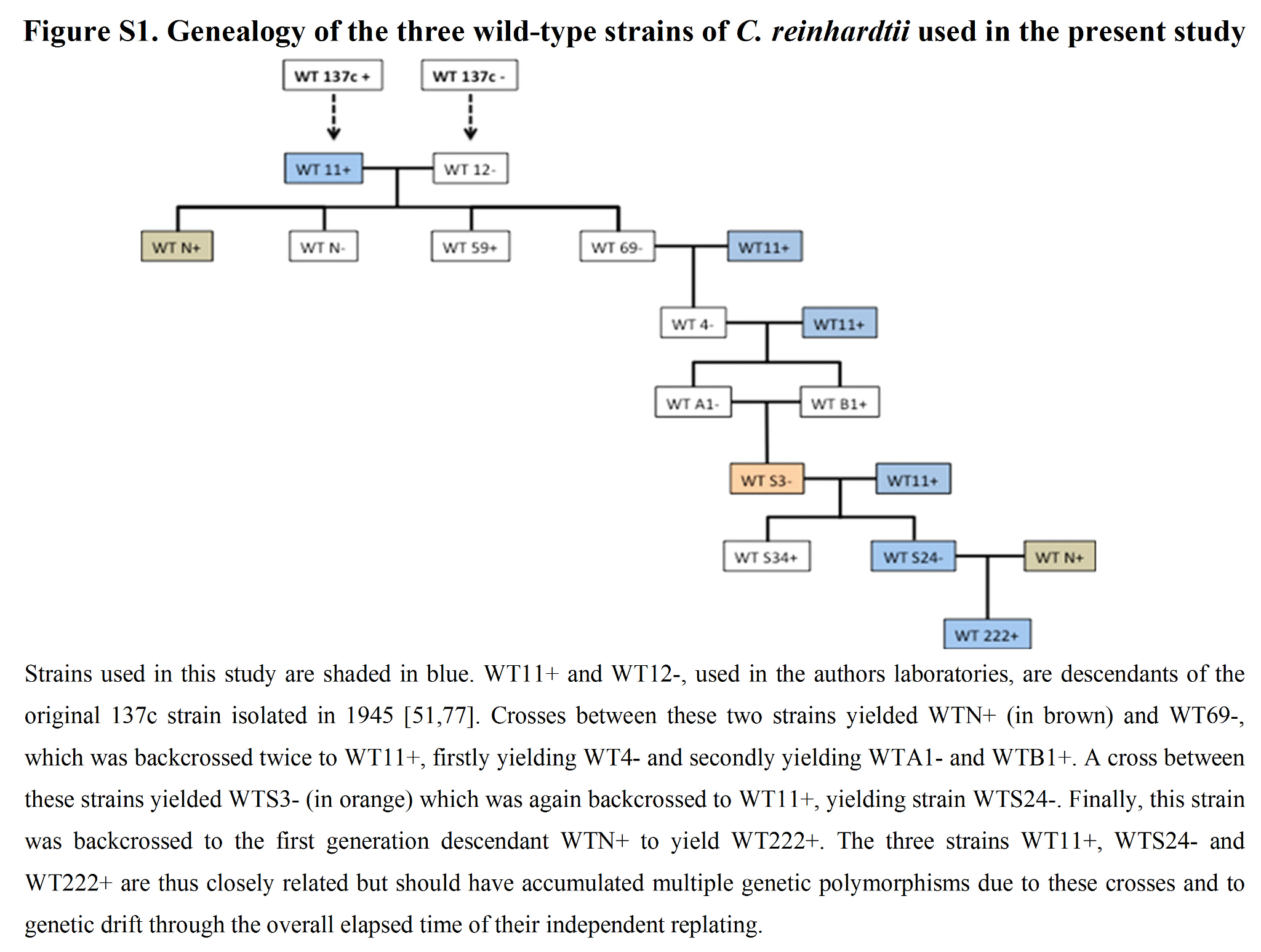

Supplement: S1 Fig — Strains used in this study are shaded in blue. WT11+ and WT12-, used in the authors laboratories, are descendants of the original 137c strain isolated in 1945 [53,79]. Crosses between these two strains yielded WTN+ (in brown) and WT69-, which was backcrossed twice to WT11+, firstly yielding WT4- and secondly yielding WTA1- and WTB1+. A cross between these strains yielded WTS3- (in orange) which was again backcrossed to WT11+, yielding strain WTS24-. Finally, this strain was backcrossed to the first generation descendant WTN+ to yield WT222+. The three strains WT11+, WTS24- and WT222+ are thus closely related but should have accumulated multiple genetic polymorphisms due to these crosses and to genetic drift through the overall elapsed time of their independent replating. (TIF) [file pone.0118987.s001.tif]

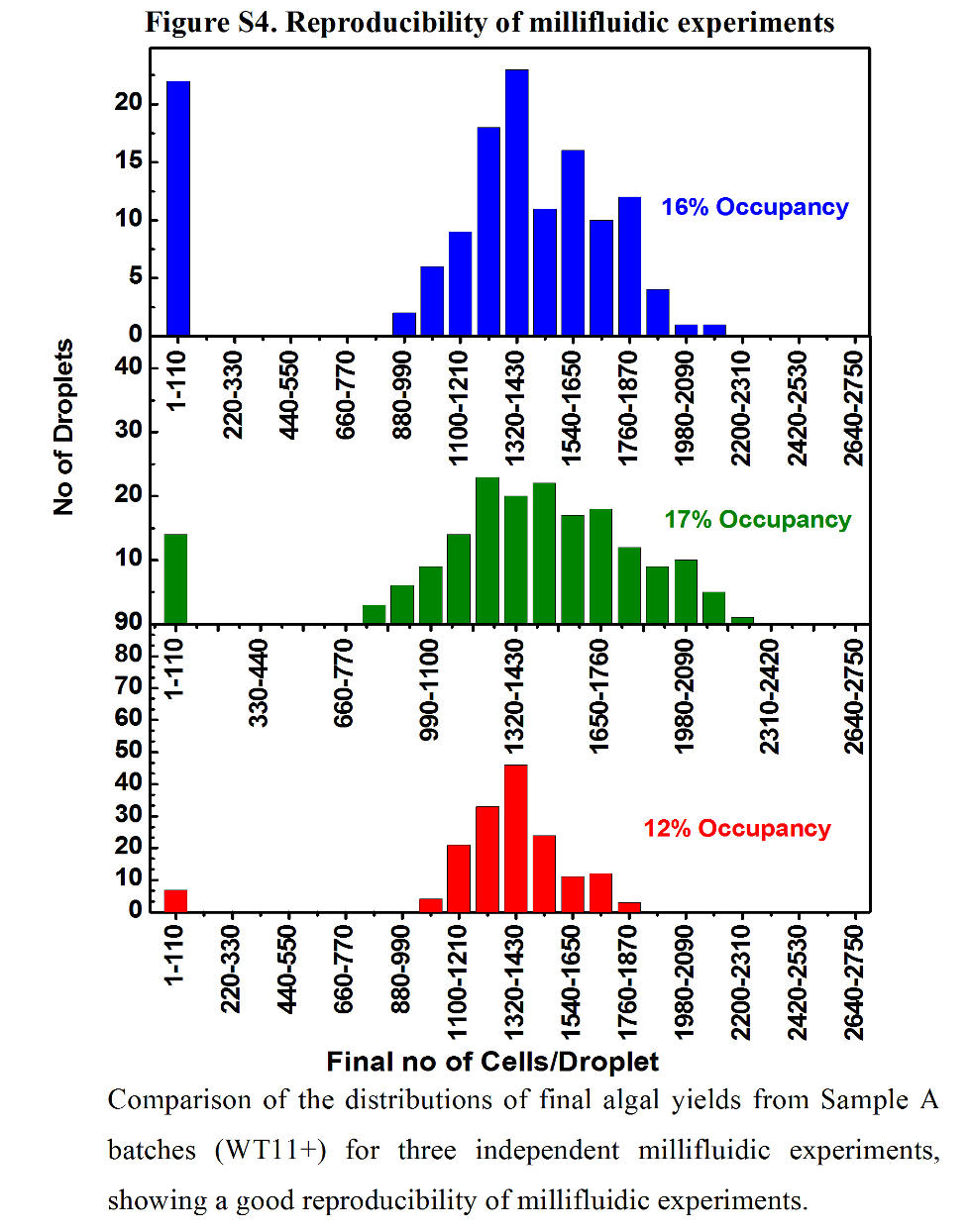

Supplement: S4 Fig — Comparison of the distributions of final algal yields from Sample A batches (WT11+) for three independent millifluidic experiments, showing a good reproducibility of millifluidic experiments. (TIF) [file pone.0118987.s004.tif]
